# Oxidative stress parameters and keap 1 variants in T2DM: Association with T2DM, diabetic neuropathy, diabetic retinopathy, and obesity

**DOI:** 10.1002/jcla.24163

**Published:** 2021-12-03

**Authors:** Farnaz Khalili, Asad Vaisi‐Raygani, Ebrahim Shakiba, Maryam Kohsari, Maryam Dehbani, Rozita Naseri, Soheila Asadi, Ziba Rahimi, Mehrali Rahimi, Zohreh Rahimi

**Affiliations:** ^1^ Department of Clinical Biochemistry Kermanshah University of Medical Sciences Kermanshah Iran; ^2^ Medical Biology Research Center Kermanshah University of Medical Sciences Kermanshah Iran; ^3^ Department of Internal Medicine Kermanshah University of Medical Sciences Kermanshah Iran

**Keywords:** diabetic neuropathy, diabetic retinopathy, keap1 variants, oxidative stress, type 2 diabetes

## Abstract

**Introduction:**

Chronic hyperglycemia activates the inflammatory pathways and oxidative stress mechanisms with consequent damage to nerve tissue and retina. The Keap1‐Nrf2 pathway acts as one of the most important antioxidant pathways of the organism. Variants of Keap1 could affect susceptibility to diabetes and its complications.

**Methods:**

In a case‐control study, 400 individuals included type 2 diabetes mellitus (T2DM) patients without complication, with neuropathy, with retinopathy, and healthy individuals were investigated. The levels of glutathione (GSH), glutathione peroxidase (GPx), malondialdehyde (MDA), and total antioxidant capacity (TAC) were measured using chemical methods. Using the PCR‐RFLP method, the Keap1 (rs11085735) variants were identified.

**Results:**

Neuropathic patients had significantly lower levels of GSH, GPx, and TAC and higher levels of total oxidative status (TOS), MDA, and oxidative stress index (OSI) compared to T2DM patients without complication and controls. Lower levels of GSH and GPx and a higher level of MDA were observed in patients with retinopathy compared with controls. Obesity was associated with significantly lower GPx activity and higher TOS. A significantly higher Keap1 AA genotype was found in patients with neuropathy than T2DM without complication and controls. The presence of Keap1 AA genotype correlated with lower GPx activity compared to CC genotype.

**Conclusions:**

Our study suggests the role of reduced antioxidant system and Keap1 variants in the pathogenesis of T2DM and its complications of neuropathy and retinopathy and also obesity in enhanced oxidative stress. Monitoring oxidative stress parameters in diabetic patients, especially those with complication and their treatment with antioxidants is suggested.

## Graphical abstract

Studying 400 T2DM patients without complication indicated: In T2DM patients without complication, the GPx and TAC levels decreased and the MDA level elevated. T2DM with neuropathy had lower levels of GSH, GPx, and TAC and higher levels of TOS, MDA, and OSI and a higher frequency of Keap1 AA genotype. T2DM with retinopathy had lower levels of GSH and GPx and a higher level of MDA. Obesity‐enhanced oxidative stress was associated with lower GPx activity and higher TOS and Keap1 AA genotype correlated with lower GPx activity. Reduced antioxidant system and Keap1 variants are involved in the pathogenesis of T2DM and its complications of neuropathy and retinopathy.

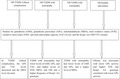

## 1 INTRODUCTION

Diabetes mellitus is a metabolic disease characterized by abnormal rising in blood glucose due to deficiency in secretion and the action of insulin.^1^ Diabetic patients have a higher risk of complications and mortality than the general population.^2^ The global prevalence of diabetes in adults has been increased in recent decades. In 2011, the prevalence of diabetes among Iranian people in the age of 25–70 years was 11.4%, indicating a 35% increased prevalence compared to that in 2005. It is estimated that by the year 2030, nearly 9.2 million of Iranian people may have diabetes.^3^

The most crucial manifestation of diabetes mellitus is vascular complications which include microvascular types such as neuropathy, retinopathy and nephropathy, and macrovascular types including peripheral arterial disease, cardiovascular disease, and stroke.^4^

Diabetic retinopathy is a serious microvascular complication of diabetes which is marked by abnormal blood flow in the retina and the inflammation of retinal blood vessels.^5^ Diabetic retinopathy, as one of the major causes of acquisitive blindness in the world, is prevalent among 41.9% of Iranian diabetic patients.^6^, ^7^

Damage to the peripheral nerves, neuropathy, is estimated to affect 10% of patients with early‐diagnosed type 2 diabetes mellitus (T2DM), and up to 50% of patients with long duration of diabetes mellitus have neuropathy.^8^ The risk of diabetic neuropathy is directly related to the duration and the severity of hyperglycemia. In addition, genetic background could affect susceptibility to diabetic neuropathy.

Free radicals and oxidative stress play a significant role in pathogenesis and progression of diabetic vascular complications. Oxidative stress is a condition induced in the body when the amount of reactive oxygen species (ROS) in cells surpasses the capacity of antioxidant.

The natural defense of human body against adverse effects of ROS is through different enzymatic and non‐enzymatic antioxidants, which regulate the production of free radicals and their metabolites. Non‐enzymatic scavengers, compounds with low molecular weight, include glutathione (GSH), ascorbic acid (vitamin C), α‐tocopherol (vitamin E), and carotenoids (β‐carotene). The most important enzymatic antioxidants that enhance and support the effect of non‐enzymatic antioxidants are the enzymes of superoxide dismutase (SOD), catalase (CAT), and glutathione peroxidase (GPx)^9^, ^10^, ^11^

The antioxidant function of Nrf2‐Keap1pathway plays a major role in protecting cells against oxidative stress.^12^ Nrf2, a member of leucine zipper family, is a transcription factor that protects cells against oxidative stress and toxicity through regulating antioxidant genes expression. The metabolic programming for response to internal and external stressors, such as ROS and environmental toxins, is directed by Nrf2.^13^, ^14^, ^15^

Keap1 (Kelch‐like ECH associated protein 1) is a cysteine‐rich protein and an excellent sensor for chemical inducers which plays an important role in regulating the Nrf2 signaling pathway. Under physiological conditions, Keap1 targets Nrf2 for proteasome degradation by its ubiquitination, thus maintaining cellular homeostasis. During oxidative stress, Nrf2 is dissociated from Keap1 and accumulated in the nucleus, where it binds to the sequence of antioxidant response elements (AREs), causing the expression of cell‐protecting genes. In this case, Keap1 acts as an important sensor for oxidative and electrophilic stress, and the high redox sensitivity of Keap1 is driven by cysteine residues that are altered by electrophiles and leading to the inactivation of Keap1, stabilization of Nrf2, and induction of many cell‐protecting genes. Some functional Keap1 mutations have been detected in various cancers and may lead to increased Nrf2/ARE gene transcription expression.^16^, ^17^ The Keap1 rs11085735 (A/C) polymorphism locates in intron 2 and intervenes with the exon sequence encodes the Kelch‐1 domain, so this polymorphism might affect the Kelch‐1 domain structure and the interaction between Keap1 and Nrf2.^18^

The present study aimed to determine possible association between Keap1 gene variants with oxidative stress parameters and with the risk of T2DM and its complications in a population from Kermanshah, Western Iran.

## 2 MATERIALS AND METHODS

### 2.1 Characteristics of patients

Studied individuals included 100 healthy individuals including 48 males and 52 females with the mean age of 52.8 ± 7.3 years, 100 patients with T2DM without complication consisted of 35 males and 65 females with the mean age of 55.9 ± 6 years, 100 T2DM patients with neuropathy (17 males and 83 females and the mean age 55.9 ± 5.8 years), and 100 T2DM patients with diabetic retinopathy, 28 males and 72 females and the mean age 58.5 ± 4.8 years, from the Diabetes Research Center of Taleghani Hospital in Kermanshah, Iran. The mean age of patients and healthy individuals was 56.8 ± 5.7 and 52.8 ± 7.3 years, respectively. None of the subjects had other systemic disease, and duration of diabetes was at least five years. The study was approved by the Ethics Committee of Kermanshah University of Medical Science, and written informed consent was obtained from each participant.

### 2.2 Sample preparation and assessment of oxidative stress parameters

Six ml blood was taken from each subject and transferred to a Falcon tube containing 0.5 mM EDTA. To test the activity of GPx, 0.5 ml of whole blood was poured in a 500 μl microtube and after plasma separation; all samples were stored in a −70°C freezer. Total antioxidant capacity (TAC), total oxidative status (TOS), lipid peroxidation index (MDA), and reduced glutathione (GSH) assays were measured in plasma. To determine the genotypes of and Keap1 (rs11085735) gene, the extraction of DNA from white blood cells was performed using phenol‐chloroform method. TAC was detected by ferric reducing ability of plasma (FRAP) method using FRAP reagent at 593 nm.^19^ TOS was measured by ferric‐xylenol orange assay using a colorimetric method.^20^ The ratio of TOS to TAC was calculated as the oxidative stress index (OSI), an indicator of the degree of oxidative stress.^21^ The presence of lipid peroxidation was determined by measuring the concentration of MDA.^22^ A convenient method using the fluorescent reagent of o‐phthalaldehyde, modified for the measurement of plasma GSH, was used for detection of GSH level at 420 nm.^23^ Glutathione peroxidase activity was determined using a commercially available kit Randox, and the activity was reported as U/mgHb.

### 2.3 Genotyping

**FIGURE 1:**
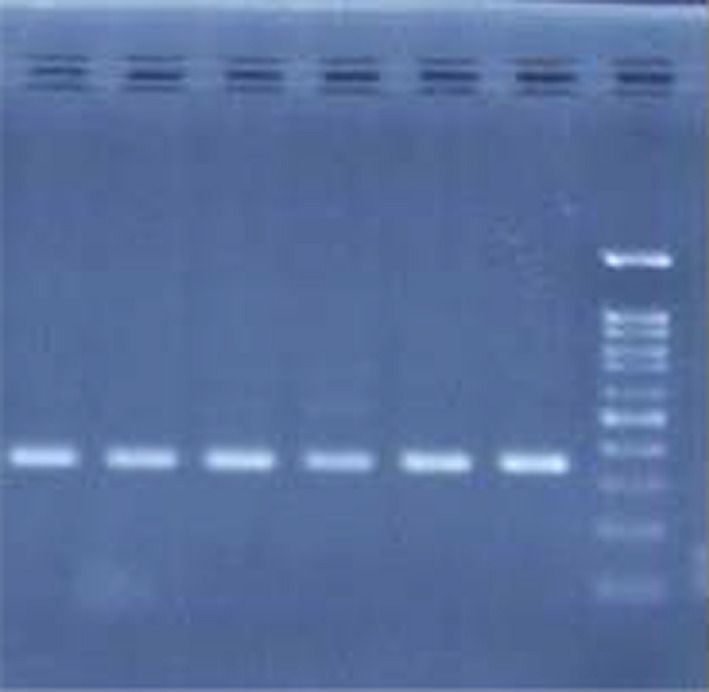
Agarose gel electrophoresis of Keap1 PCR products with the fragment size of 354‐bp

**FIGURE 2:**
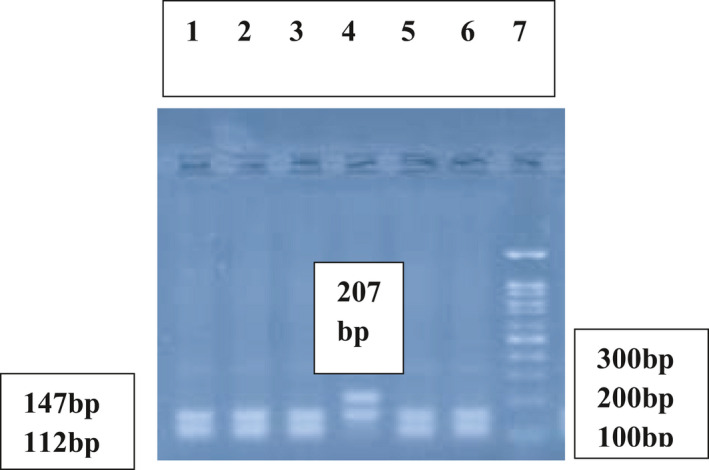
Agarose gel electrophoresis pattern of digested PCR products with the HinfI restriction enzyme. Lanes 1, 2, 3, 5, 6 indicate the CC genotype. Lane 4 demonstrates the AA genotype. Lane 7 depicts 100‐bp DNA molecular weight marker

DNA was extracted from EDTA‐treated whole blood.^24^ The DNA content of each sample was quantified and its purity assessed with a NanoDrop spectrophotometer. Detection of Keap1 (rs11085735) (A/C) polymorphism was performed using polymerase chain reaction (PCR) and subsequent digestion of PCR products with HinfI restriction enzyme. The designed specific Keap1 primers were 5ˊ CTC AGC CTC CCA AAG TCC CT 3ˊ(forward) and 5ˊ CTC CCA CGG CTG CAT CCA C 3ˊ(reverse). Thermal cycling process in PCR was initial denaturation at 94°C for 5 min; 35 cycles of 94 C for 30 sec, 63°Cfor 30 sec, 72°C for 30 sec; and a final elongation at 72°C for 10 min. The obtained PCR product with the fragment size of 354‐bp was digested with HinfI restriction enzyme. In the presence of AA genotype, two fragments with 207‐ and 147‐bp were obtained. However, in the presence of CC genotype, the 207‐bp fragment was digested by HinfI to two fragments of 112‐ and 95‐bp and three fragments were detected (147‐, 112‐, and 95‐bp). In the presence of AC genotype four fragments of 207‐, 147‐, 112‐, and 95‐bp were obtained (Figures 1 and 2).

### 2.4 Statistical analysis

Statistical analysis was done using SPSS software package version 16, and the results were extracted. Comparison of the Keap1 genotypes frequency was performed among four studied groups using χ^2^ test and the Pearson correlation coefficient. To investigate the relationship between plasma levels of quantitative parameters among four groups, t test and ANOVA were used. The *p* < 0.05 was considered as the significant level.

## 3 RESULTS

### 3.1 Demographic Characteristics and oxidative stress parameters

Characteristics of studied individuals are demonstrated in Table 1. Diabetic patients without complication had the BMI level 28.2 ± 3.4 Kg/m^2^ that was not significantly different from controls (26.9 ± 4.1 Kg/m^2^, *p*=0.15). However, diabetic patients with neuropathy had significantly higher BMI level (30.5 ± 4.7 Kg/m^2^) compared to controls (*p* < 0.001), diabetic patients without complication (*p* = 0.001), and diabetic retinopathy patients (*p* = 0.004). Also, the BMI level of patients with diabetic retinopathy (28.5 ± 4.3 Kg/m^2^, *p* = 0.046) was significantly higher than controls. The levels of HbA_1c_ were significantly higher in diabetic retinopathy (9.6 ± 6.2%, *p* = 0.028) and diabetic neuropathy patients (8.8 ± 1.8%, *p* = 0.013) compared to diabetic patients without complication (8.1 ± 1.6%) (Table 1). Among diabetic neuropathy patients, there were 88 (88%) patients with BMI>25 Kg/m^2^ that their TOS level was significantly higher (4.3 ± 2.2 nmol/ml, *p* = 0.041) compared to those normal weight neuropathic patients with BMI≤25 Kg/m^2^ (3.5 ± 0.96 nmol/ml). In diabetic retinopathy patients, there were 80 (80%) patients with BMI>25 Kg/m^2^ that their TOS level was significantly higher (3.9 ± 2.7 nmol/ml, *p* = 0.02) compared to those normal weight patients with BMI≤25 Kg/m^2^ (3.1 ± 0.76 nmol/ml). In obese diabetic neuropathy patients (BMI>30 Kg/m^2^), the MDA level was significantly higher (7.6 ± 2.2 nmol/ml) compared to non‐obese patients, BMI≤30 Kg/m^2^ (6.7 ± 2.2 nmol/ml, *p* = 0.048). T2DM patients without complication had lower level of TAC (731.6 ± 124.1 nmol/ml) and GPx activity (51.3 ± 11.3 U/mgHb) than control group (770.2 ± 113.7 nmol/ml, *p* = 0.023, and 59.9 ± 10.9 U/mgHb, *p* < 0.001, respectively). However, the level of MDA was significantly higher in these patients (6.6 ± 1.9 nmol/ml) than controls (5.1 ± 1.6 nmol/ml, *p* < 0.001) (Table 1).

**TABLE 1:** Characteristics of patients and controls

Variables	T2DM with retinopathy (*n* = 100) Mean ± SD	T2DM with neuropathy (*n* = 100) Mean ± SD	T2DM without complication (*n* = 100) Mean ± SD	Controls (*n* = 100) Mean ± SD
Age (years) Range	58.5 ± 4.8	55.9 ± 5.8	55.9 ± 6.0	7.3 ± 52.8
	46–67 *p* < 0.001^a^, *p* = 0.017^b^, ^c^p = 0.017	45–65 *p* = 0.001^a^, *p* = 0.99^b^	45–65 *p* = 0.001^a^	45–65
BMI (Kg/m^2^)	28.5 ± 4.3	30.5 ± 4.7	28.2 ± 3.4	26.9 ± 4.1
	*p* = 0.046^a^, *p* = 0.004^c^	*p* < 0.001^a^, *p* = 0.001^b^	*p* = 0.15^a^	
Diabetes duration (years)	15.5 ± 5.4 *p* < 0.001^b^, *p* < 0.001^c^	10.6 ± 4.4 *p* = 0.98^b^	10.7 ± 4.4	–
HbA_1c_	9.6 ± 6.2	8.8 ± 1.8	8.1 ± 1.6	–
(%)	*p* = 0.028^b^, *p* = 0.31^c^	*p* = 0.013^b^		
Glutathione	9.6 ± 3.9	7.8 ± 4.6	12.2 ± 2.5	11.3 ± 3.4
(nmol/ml)	*p* = 0.004^a^, *p* < 0.001^b^, *p* = 0.004^c^	*p*<0.001^a^, *p* < 0.001^b^	*p* = 0.029^a^	
GPx	48.2 ± 7.7	39.6 ± 8.5	51.3 ± 11.3	59.9 ± 10.9
(U/mgHb)	*p* < 0.001^a^, *p* = 0.1^b^, *p* < 0.001^c^	*p* < 0.001^a^, *p* < 0.001^b^	*p* < 0.001^a^	
TAC	793.5 ± 124.7	703.2 ± 129.2	731.6 ± 124.1	770.2 ± 113. 7
(nmol/ml)	*p* = 0.54^a^, *p* = 0.002^b^, *p* < 0.001^c^	*p* < 0.001^a^, *p* = 0.011^b^	*p* = 0.023^a^	
TOS	3.7 ± 2.4	4.2 ± 2.1	3.5 ± 1.4	4.2 ± 1.2
(nmol/ml)	*p* = 0.32^a^, *p* = 0.73^b^, *p* = 0.28^c^	*p* = 0.94^a^, *p* = 0.004^b^	*p* < 0.001^a^	
MDA	6.7 ± 2.0	7.2 ± 2.3	6.6 ± 1.9	5.1 ± 1.6
(nmol/ml)	*p* < 0.001^a^, *p* = 0.99^b^, *p* = 0.22^c^	*p* < 0.001^a^, *p* = 0.050^b^	*p* < 0.001^a^	
OSI	0.0047 ± 0.0029	0.0062 ± 0.0032	0.0048 ± 0.002	0.0055 ± 0.0017
	*p* = 0.13^a^, *p* = 0.99^b^, *p* = 0.001^c^	*p* = 0.076^a^, *p* = 0.001^b^	*p* = 0.012^a^	

^a^ Comparisons were made with controls.

^b^ Comparisons were made with T2DM patients without complication.

^c^ Comparison were made with T2DM patients with neuropathy

As indicated in Table 1, T2DM patients with neuropathy had lower levels of GSH (7.8 ± 4.6 nmol/ml), TAC (703.2 ± 129.2 nmol/ml) and GPx activity (39.6 ± 8.5 U/mgHb) than control group (11.3 ± 3.4 nmol/ml, *p* < 0.001; 770.2 ± 113.7 nmol/ml, *p* < 0.001, and 59.9 ± 10.9 U/mgHb, *p* < 0.001, respectively) and also compared to T2DM patients without complication (12.2 ± 2.5 nmol/ml, *p* = 0.029; 51.3 ± 11.3 U/mgHb, *p* < 0.001; and 731.6 ± 124.1 nmol/ml, *p* = 0.023, respectively). Analyzing oxidative stress parameters in both genders indicated T2DM patients had significantly lower level of GSH (10.2 ± 3.9 vs. 11.6 ± 3.5, *p* = 0.04 in males and 9.8 ± 4.2, vs. 11.1 ± 3.2 nmol/ml, *p* = 0.013) and GPx activity (58.6 ± 10.2, vs. 48.3 ± 11.7, *p* < 0.001 in males and 45.7 ± 10, vs. 61.1 ± 11.4 U/mgHb, *p* < 0.001 in females) and higher level of MDA (6.5 ± 2.1 in males, vs. 5.1 ± 1.6, *p* < 0.001 and 6.9 ± 2.1, vs. 5 ± 1.7 nmol/ml, *p* < 0.001 in females) in both genders compared to controls. Also, TOS level was significantly higher in neuropathic patients (4.2 ± 2.1 nmol/ml) than T2DM patients without complication (3.5 ± 1.4 nmol/ml, *p* = 0.004) and OSI was significantly higher in these patients (0.0062 ± 0.0032) than T2DM patients without complication (0.0048 ± 0.0021, *p* = 0.001). Further, the level of MDA was significantly higher in diabetic neuropathy patients (7.2 ± 2.3 nmol/ml) compared to controls (5.1 ± 1.6 nmol/ml, *p* < 0.001).

The level of GSH was significantly lower in T2DM patients with retinopathy (9.6 ± 3.9 nmol/ml) in comparison with control group (11.3 ± 3.4 nmol/ml, *p* = 0.004) and also comparing with T2DM patients without complication (12.2 ± 2.5, *p* < 0.001). Significantly lower level of GPx (48.2 ± 7.7 U/mgHb) and higher level of MDA (6.7 ± 2.0 nmol/ml) in patients with retinopathy compared to control group (59.9 ± 10.9 U/mgHb, *p* < 0.001; and 5.1 ± 1.6, *p* < 0.001, respectively) was observed. Also, T2DM patients with diabetic retinopathy had higher GSH (9.6 ± 3.9 nmol/ml), and TAC (793.5 ± 124.7 nmol/ml) levels and GPx activity (48.2 ± 7.7 U/mgHb) than those with diabetic neuropathy (7.8 ± 4.6 nmol/ml, *p* = 0.004; 703.2 ± 129.2 nmol/ml, *p* < 0.001; and 39.6 ± 8.5 U/mgHb, *p* < 0.001, respectively). However, OSI was significantly lower in patients with diabetic retinopathy (0.0047 ± 0.0029) compared to those with diabetic neuropathy (0.0062 ± 0.0032, *p* = 0.001) (Table 1).

### 3.2 Allele and genotype distribution of Keap1 and its relation with oxidative parameters

Distribution of Keap1 genotypes and alleles among T2DM patients without complication, T2DM patients with neuropathy, T2DM patients with retinopathy and controls are presented in Table 2. The frequency of AA genotype was 4% in diabetic neuropathy patients compared to 0% in T2DM patients without complication (0%, *p* = 0.049) and 0% in controls (*p* = 0.048).

**TABLE 2:** Distribution of Keap1 genotypes and alleles in patients and controls

Keap1 parameters		T2DM with neuropathy *n* (%)	T2DM with retinopathy *n* (%)	T2DM without complication *n* (%)	Controls *n* (%)
Genotypes	AA	4	0	0	0
	AC	4	20	9	8
	CC	92	80	91	92
		χ^2^ = 3.9^a^, *p* = 0.048 χ^2^ = 3.88^b^, *p* = 0.049			
Alleles	A	12 (6)	20 (10)	9 (4.5)	8 (4)
	C	188 (94)	180 (90)	191 (95.5)	192 (96)
		χ^2^ = 0.84^c^, *p* = 0.36 χ^2^ = 0.45^d^, *p* = 0.50	χ^2^ = 5.5^c^, *p* = 0.019 OR = 2.67, [95%CI (1.2–6.2), *p* = 0.023] χ^2^ = 4.5^d^, *p* = 0.034 OR = 2.36, [95%CI (1.05–5.32, *p* = 0.039]	χ^2^ = 0.061^a^, *p* = 0.8	

^a^ Comparing AA with CC genotype between patients and controls.

^b^ Comparing AA with CC genotype between neuropathic patients and diabetic patients without complication.

^c^ Comparisons were made with controls.

^d^ Comparisons were made with T2DM patients without complication.

Higher frequency of A allele of Keap1 (10%) was found in T2DM patients with retinopathy compared to T2DM patients without complication (4.5%, *p* = 0.034, OR = 2.36) and controls (4%, *p* = 0.019, OR = 2.67). Higher frequency of minor A allele in T2DM with neuropathy (6%) compared to those in controls and T2DM without complication did not reach to a statistical significant (Table 2).

Oxidative stress biomarkers levels in relation to genotypes of Keap1 among all studied individuals are demonstrated in Table 3. Carriers of Keap1 AA genotype compared to those carrying CC genotype had significantly lower GPx activity (37.2 ± 5 vs. 49.7 ± 12.3 U/mgHb, *p* = 0.01). However, carriers of CC genotype had higher OSI level than those with heterozygote genotype of AC (*p* = 0.03) (Table 3). Oxidative stress parameters between various genotypes of Keap1 in patients with diabetic neuropathy and also in patients with diabetic retinopathy were analyzed separately, and no significant difference was found comparing AC and CC genotypes with AA genotype. Oxidative stress parameters in different genotypes of Keap1 in both diabetic neuropathy and retinopathy are presented in Table 4 and have been compared between both groups. The MDA level and OSI were significantly higher in Keap1 CC genotype of neuropathic patients than patients with retinopathy (Table 4).

**TABLE 3:** Association of oxidative parameters with genotypes of Keap1 in all studied individuals

Variable	AA Mean ± SD	AC Mean ± SD	CC Mean ± SD	*p* value (AA vs. CC)	*p* value (AC vs. CC)
Glutathion (nmol/ml)	7.2 ± 5.4	9.8 ± 3.9	10.3 ± 4.0	*p* = 0.12	*p* = 0.41
GPx (U/mgHb)	37.2 ± 5	51.8 ± 9.9	49.7 ± 12.3	*p* = 0.01	*p* = 0.21
TAC (nmol/ml)	760.8 ± 56.7	753.3 ± 107.7	749.1 ± 130.2	*p* = 0.71	*p* = 0.82
MDA (nmol/ml)	8 ± 1.7	6.7 ± 2.3	6.3 ± 2.1	*p* = 0.11	*p* = 0.33
TOS (nmol/ml)	4.1 ± 0.8	3.5 ± 1.5	3.9 ± 1.9	*p* = 0.75	*p* = 0.07
OSI	0.0055 ± 0.0015	0.0047 ± 0.0019	0.0054 ± 0.0027	*p* = 0.93	*p* = 0.03

**TABLE 4:** Comparing oxidative stress parameters in various genotypes of Keap1 in diabetic patients with neuropathy and retinopathy

Oxidative Stress arameters								
	Neuropathy AA Mean ± SD	Retinopathy A A	Neuropathy AC Mean ± SD	Retinopathy AC Mean ± SD	*p* value	Neuropathy CC Mean ± SD	Retinopathy CC Mean ± SD	*p* value
Glutathione (nmol/ml)	7.21 ± 5.43	–	6.1 ± 2.6	9.2 ± 4.2	0.9	7.9 ± 4.6	9.6 ± 3.7	0.9
GPx (U/mgHb)	37.22 ± 5.01	–	42.6 ± 12.5	49.8 ± 7.7	0.8	39.5 ± 8.4	47.7 ± 7.6	1.0
TAC (nmol/ml)	760.8 ± 56.69	–	697.3 ± 108.2	801 ± 95.0	0.9	700.8 ± 132.3	791.5 ± 131.4	1.0
TOS (nmol/ml)	4.10 ± 0.83	–	2.9 ± 0.8	3.6 ± 1.8	0.7	4.2 ± 2.1	3.7 ± 2.5	0.07
MDA (nmol/ml)	8 ± 1.70	–	8.4 ± 3.2	7.0 ± 2.5	0.1	7.1 ± 2.2	6.5 ± 1.8	0.04
OSI	0.005 ± 0.001	–	0.004 ± 0.001	0.004 ± 0.002	0.6	0.006 ± 0.003	0.004 ± 0.003	< 0.001

*p* values comparing all parameters between three genotypes in patients with diabetic neuropathy were>0.05.

*p* values comparing all parameters between two genotypes in patients with diabetic retinopathy were>0.05.

## 4 DISCUSSION

Oxidative stress is involved in the pathogenesis of diabetes and its complications. Oxidative stress is often known as an imbalance between the levels of ROS and antioxidants that may be induced by altered gene expression. Nrf2 transcription factor is the main regulator of the antioxidant response.^25^ The antioxidant function of Nrf2‐Keap1 pathway has a major role in cell protection against oxidative stress.^12^

The present study has focused on oxidative stress‐related parameters and Keap1 rs11085735 variants in T2DM and its complications, diabetic neuropathy and diabetic retinopathy, and possible association between Keap1 variants with oxidative stress parameters, T2DM and the development of neuropathy and retinopathy in T2DM patients.

T2DM without complication.

Among T2DM patients without complication of present study, the GPx activity and TAC level were significantly decreased and MDA level was significantly elevated compared to those in healthy individuals. Our findings indicated low GPx activity could be a risk factor for T2DM. Decreased GPx activity in hyperglycemia might be result from protein glycation, which is a mechanism that damages the protein of antioxidant enzymes.^26^ However, it has been reported that the erythrocyte GPx activity was significantly higher in diabetic and pre‐diabetic groups in comparison with controls. Diabetic subjects with poor glycemic control showed the highest GPx among all studied groups.^27^

Oxidative stress is involved in insulin resistance and dysfunction of β cells through dysregulation of cell homeostasis and metabolism. Negative correlation between TAC level and glycemic level has been indicated as with increased hyperglycemia in T2DM, the plasma antioxidant capacity is worsening. However, there are inconsistent results as an increased TAC levels in these patients have been reported in some studies.^28^

Enhanced MDA level is an index of increased lipid peroxidation in diabetic patients. Elevated MDA level has adverse physiological consequences of cell membrane structural integrity alteration, inactivating membrane bound enzymes, and cell surface receptors.^28^

T2DM with neuropathy.

We found antioxidant parameters, GSH and TAC levels and GPx activity, significantly decreased in T2DM with neuropathy compared to those diabetic patients without complication and controls and reduced levels of these antioxidant agents could be a risk factor for diabetic neuropathy. However, there are contradictory reports about the status of antioxidant activity in diabetes and both increased and decreased antioxidant parameters have been reported.^29^, ^30^ GSH is the most abundant cell antioxidant that plays an essential role in antioxidant defense by regulating redox state and neutralizing free radicals. Hyperglycemia status indirectly results in GSH depletion through the polyol pathway, which consumes NADPH for GSH regeneration through reaction catalysis by glutathione reductase.^31^

In the present study, obesity was associated with significantly lower GPx activity and higher TOS considering all diabetic patients. Also, the MDA level was significantly higher comparing obese diabetic neuropathy patients with non‐obese ones. Our findings suggest obesity in T2DM is associated with increased oxidative stress that is reflected in reduced GPx activity, higher TOS level and increased lipid peroxidation.^32^

Dyslipidemia due to obesity induces overproduction of non‐esterified fatty acids by β‐oxidation, this process along with biosynthesis of deoxy‐sphingolipids, causes reduction of ATP production and ROS overload, which activates inflammatory signals that lead to mitochondrial dysfunction, endoplasmic reticulum stress, DNA damage, and apoptosis in cells of nervous system.^33^ Among obese and centrally obese T2DM the GPx activity and plasma MDA increased and the SOD activity decreased indicating obesity and central obesity enhances the oxidative stress in T2DM.^34^

A considerable point in the present study was a significantly different duration of diabetes between retinopathy and neuropathy patients. Retinopathy patients had higher diabetes duration and also higher Hb A_1c_ but lower BMI level than neuropathy subjects. Oxidative condition is expected to be worsening in these patients than in patients with neuropathy, but the results were reversed, and neuropathic patients had lower antioxidants and higher oxidants parameters. Smith and Singleton based on intra‐epidermal nerve fiber density suggested obesity and triglycerides were related to loss of small unmyelinated axons and obesity could be an early diabetic neuropathy risk factor, independent of glycemic control.^35^ On the other hand, in a study, among 420 diabetic patients in Singapore increased BMI inversely correlated with the risk of retinopathy in T2DM women and the study hypothesized which BMI could have a protective effect on diabetic retinopathy.^36^

The present study detected a significantly higher frequency of minor Keap1 AA genotype in T2DM with neuropathy compared to those diabetic patients without complication and also compared to controls. The Nrf2‐Keap1 pathway is an important mechanism against oxidative stress. According to the literature, there is no available report related to the frequency of Keap1 variants in T2DM and its complications of retinopathy and neuropathy and their influence on the risk of T2DM and its complications.

The Keap1 mutation has a role in the development and progression of various human cancers.^37^ The minor A allele of Keap1 rs11085735 has been associated with lower Keap1 and higher nuclear Nrf2 expression and decreased overall survival in breast cancer patients treated with radiotherapy and tamoxifen.^18^ Keap1‐Nrf2 pathway protects beta cells of pancreas against oxidative stress and is involved in the maintenance of glucose metabolism through both insulin secretion and utilization of glucose in insulin‐sensitive tissues.^38^ Tissue regeneration models indicated an association between hyperglycemia in diabetes with dysfunction of Keap1 through preventing nuclear localization of Nrf2.^39^ The Nrf2 has a dual role in the pathogenesis of diabetes and obesity. Some reports indicated hyperglycemia stimulated the polyol pathway and raised the protein kinase C activity and NF‐κB. These changes through the oxidative stress resulted in increased Nrf2 expression. Overexpression of Nrf2 (Keap1 knockdown) can act as a protective role in diabetes complications such as neuropathy.^40^ In contrast with these findings, various studies have demonstrated increased Nrf2 expression aggravated the phenotypes of obesity and insulin resistance. These reports were heavily dependent on the analyses of Nrf2 knockout mice.^41^, ^42^ As Chen et al. in a study on diabetes‐induced rats, by using natural rotenoid deguelin ameliorated diabetic neuropathy with remarkably repressed mechanical and thermal hyperalgesia and slightly restored the conduction velocities of neurons in diabetic neuropathy rats through the inhibition of Nrf2 and NF‐κB pathways.^43^ Our results showed that neuropathy patients had a higher frequency of minor genotype of AA compared to retinopathy patients and individuals without the complication that might results in increased Nrf2 and decreased Keap1 contributing to diabetic neuropathy.

### 4.1 Diabetic retinopathy

In diabetic retinopathy, the presence of large amounts of ROS results in change and damage of micro‐vessel, cells, and ganglion cells of retinal. Oxidative stress is a major pathogenic mechanism of diabetic retinopathy. Hyperglycemia or diabetes mellitus can potentiate oxidative stress in organism. In a rat model of T2DM with retinopathy elevated cell apoptosis and ROS and also increased MDA content in retinal tissues along with remarkably decreased antioxidant potency has been reported. Also, the study detected lower Keap1 and higher Nrf2 expression in retinal tissues and demonstrated the initiation of endogenous oxidative stress system in diabetic rat retina. The oxidative stress level was significantly higher in patients with diabetic retinopathy than T2DM without complication and also compared to controls.^44^ In the present study, the level of GSH and the activity of GPx were significantly lower in diabetic retinopathy patients compared with T2DM patients without complication and controls. However, a significantly higher level of MDA in diabetic retinopathy patients than controls was observed. MDA is a most common index for evaluation of body oxidative stress and lipid peroxidation status.

The Keap1‐Nrf2 pathway in the streptozotocin‐induced diabetic model had a protection role against the onset and/or diabetic retinopathy progression.^38^ The presence of Keap1 rs11085735 (A/C) polymorphism, the minor allele A, was associated with lower expression of KEAP1 protein and elevated nuclear Nrf2 expression.^18^ The minor Keap1 A allele was significantly more frequent in T2DM with retinopathy than T2DM patients without complication and controls. Our study indicated the Keap1 A allele increased the risk of diabetic retinopathy by 2.36‐fold. Also, the presence of this allele increased the risk of T2DM with complication of retinopathy by 2.67 times. Lower Keap1 and higher Nrf2 expression in diabetic model retinal tissues demonstrated initiation of endogenous oxidative stress system in retina.^44^ Our findings indicate the role of oxidative stress along with overweight, through increased TOS level, and also Keap1 polymorphism in diabetic retinopathy pathogenesis as the minor A allele of Keap1 was associated with lower Keap1 expression^18^ and also the presence of Keap1 AA genotype was correlated with lower GPx activity than CC genotype in our study.

## 5 CONCLUSION

Among T2DM patients without complication, the GPx activity and TAC level were significantly decreased and MDA level was significantly elevated compared to those in healthy individuals. Also, we found antioxidant parameters of GSH and TAC levels and GPx activity significantly decreased in T2DM with neuropathy compared to those diabetic patients without complication and controls. In the present study, obesity was associated with significantly lower GPx activity and higher TOS considering all diabetic patients. Also, the MDA level was significantly higher comparing obese diabetic neuropathy patients with non‐obese ones. Our findings suggest hyperglycemia status results in GSH depletion and obesity in T2DM is associated with increased oxidative stress that is reflected in reduced GPx activity, higher TOS level and increased lipid peroxidation. Further, the present study detected a significantly higher frequency of AA genotype in T2DM with neuropathy compared to those diabetic patients without complication and also compared to controls that might result in increased Nrf2 and decreased Keap1 expression contributing to diabetic neuropathy. In the present study, the oxidative stress level was significantly higher in patients with diabetic retinopathy than T2DM without complication and also compared to controls. Our findings indicate the role of oxidative stress along with overweight, through increased TOS level, and also Keap1 polymorphism in diabetic retinopathy pathogenesis as the minor A allele of Keap1 is associated with lower Keap1 expression. Furthermore, the presence of Keap1 AA genotype correlated with lower GPx activity compared to CC genotype. Given that studies in this field have been performed on animal models and no results have been obtained in humans, more studies are needed, especially related to Keap1 variants and the risk of development of diabetes and its complications. Our study suggests monitoring of diabetic patients especially those with complication for the oxidative stress parameters and possible treatment with antioxidants.

### 5.1 Limitation of the study

The limitation of the present study was lower number of studied males in patients compared to healthy individuals.

## CONFLICTS OF INTEREST

None.

## Data Availability

The datasets used and/or analyzed during the current study are available from the corresponding author on reasonable request.
